# DOK5 as a Prognostic Biomarker of Gastric Cancer Immunoinvasion: A Bioinformatics Analysis

**DOI:** 10.1155/2022/9914778

**Published:** 2022-01-05

**Authors:** Fengyong Luo, Zhihuai Wang, Shuai Chen, Zhenbo Luo, Gaochao Wang, Haojun Yang, Liming Tang

**Affiliations:** ^1^School of Graduate, Dalian Medical University, Dalian, Liaoning 116044, China; ^2^Center of Gastrointestinal Disease, The Affiliated Changzhou No. 2 People's Hospital of Nanjing Medical University, Changzhou, Jiangsu 213000, China

## Abstract

**Background:**

Docking protein 5 (DOK5) is a member of the docking protein group of membrane proteins and is an adapter protein involved in signal transduction. Nevertheless, the role of DOK5 expression in the prognosis of gastric cancer (GC) remains unclear.

**Methods:**

In this study, clinical prognostic parameters and survival data related to DOK5, in patients with GC, were analyzed using bioinformatics analysis comprising Oncomine and TIMER, UALCAN database, Kaplan-Meier plotter, GEPIA, GSEA, DAVID, and cBioPortal websites.

**Results:**

In our study, GC contained various DOK5 expressions, which forecasted poor survival outcomes. Moreover, our research showed that high DOK5 could predict high-level infiltration of several GC immune cells, as evidenced by M1, TAM, M2, B cell, and T cell failure. Hence, DOK5 might become a new gastric cancer biomarker and therapeutic target. In the following analysis, in order to explore the prognostic value of DOK5 in GC, more clinical trials are needed to validate our results.

**Conclusions:**

Through multiple database verifications, DOK5 was found to be part of the pathogenic genes for GC. Thus, it can change the formation and progression of tumors by acting on human immunity.

## 1. Introduction

Gastric cancer (GC) is globally significant. It is the 5th most diagnosed cancer and 3rd major cause of cancer-related deaths. It is twice as common in men than in women [1]. GC is deemed a high-mortality cancer due to delayed diagnosis, as no certain clinical symptom appears during the early stage [2]. Hence, exploring certain sensitive biomarkers is highly important for early diagnosis as well as the prognostic evaluation of patients with GC.

Docking protein 5 (*DOK5*), which was first reported in 2001, is a member of a subgroup of the DOK family that has been expressed using c-Ret in several neuronal tissues. The receptor, tyrosine kinase c-Ret, had been explored as an oncogene, which also has been mutated in patients with multiple endocrine neoplasia and familial medullary thyroid cancer syndromes. *DOK5* enhances c-Ret-dependent activation of mitogen-activated protein kinase [3]. Favre et al. had shown that *DOK5* are expressed in T cells and their expression is regulated upon T cell activation [4]. Pothlichet et al. suggest that *DOK5* upregulation might also be associated with metastasis, in human melanoma [5].

The above findings indicate that *DOK5* plays a key role in the invasion, progression, and the metastasis of cancer. In this study, we systematically assessed *DOK5* expression in a variety of tumor forms involving GC, as well as its association with prognosis. We also assessed its status regarding distinct tumor-infiltrating immune cells, using the Oncomine database, TIMER databases, GEPIA, Kaplan-Meier plotter, and UALCAN database. Our results clarified the significant role of *DOK5* in the prognosis of GC and offer a potential mechanism by which *DOK5* expression might monitor tumor immunity—regulation of the infiltration of immune cells in GC.

## 2. Methods and Materials

### 2.1. Oncomine

The *DOK5* GC and normal tissues' mRNA expression levels were checked using the Oncomine database [website address (https://www.oncomine.org/resource/login.html)]. We selected *P* value = 1*E* − 4; two-fold change in the study and top 10% gene rank had been utilized for the threshold. Wang's studies were used to assess the differential expression levels of GC genes.

### 2.2. GEPIA

Gene Expression Profiling Interactive Analysis (GEPIA) (http://gepia.cancer-pku.cn/) is a modern web-based tool containing data on gene expression in normal tissues and tumors, shared from TCGA (The Cancer Genome Atlas), as well as the Genotype-Tissue Expression project; thus, it implements a standard processing pipeline [6]. It gives optional functions like differential expression analysis in tumors as well as normal tissues. We could also illustrate *DOK5* expression in GC, as well as normal tissues.

### 2.3. TIMER

The Tumor Immune Estimation Resource (TIMER) platform has been used to assess the tumor-infiltrating immune cells of 32 cancer types in a comprehensive way. It utilized 10,000+ samples from TCGA platform (https://cistrome.shinyapps.io/timer/). TIMER assesses a mass of tumor-infiltrating immune cells using the statistical analysis of the gene expression profiles [7]. We examined the link shared by the *DOK5* gene expression level along with the abundance of infiltrating immune cells, comprising CD8+ T cells, CD4+ T cells, neutrophils, B cells, and macrophages and dendritic cells based on the expression regarding marker genes in various cancers involving GC. Those marker genes utilized the analysis of the tumor-infiltrating immune cells involving B cells, T cells, monocytes, TAMs, M2 and M1 macrophages, natural killer (NK) cells, neutrophils, dendritic cells (DCs), T-helper 17 (Th17) cells, T-helper (Th) cells, exhausted T cells, and follicular helper T (Tfh) cells, along with Tregs that had been based on data taken from past researches. *DOK5* gene was on the *x*-axis, and related marker genes were on the *y*-axis.

### 2.4. Kaplan-Meier Plotter

The Kaplan-Meier plotter platform (https://kmplot.com/analysis/) integrates information from TCGA, EGA, and GEO databases and translates the impact of target genes on patients with cancer. To evaluate the impact of *DOK5* on the prognosis of patients with GC, Kaplan-Meier plotter was used, using various pathological parameters.

### 2.5. UALCAN

The UALCAN platform (http://ualcan.path.uab.edu) makes use of RNA-seq as well as the clinical data of 31 different cancer categories through TCGA [8]. It is capable of analyzing the tumor and normal sample's relative gene expression, at varying tumor stages, tumor grades, and other clinicopathological features.

### 2.6. Functional Enrichment Analyses of Gastric Cancer

We ran Kyoto Encyclopedia of Genes and Genomes (KEGG) as well as Gene Ontology (GO) functional enrichment assessment on *DOK5*. The Database for Annotation, Visualization and Integrated Discovery (DAVID, https://david.ncifcrf.gov/) was utilized for the identification of enriched pathways as well as terms of GO and KEGG.

### 2.7. Gene Set Enrichment Analysis

We used the Perl software to compile the expression dataset file along with the phenotype data file of the target gene for the single gene enrichment analysis. We downloaded and installed the GSEA software (http://software.broadinstitute.org/ gsea) and ran it in a Java8 environment. The target gene was enriched by KEGG pathway analysis, and the path for analysis was obtained through the c2.cp.kegg.v7.2.symbols.gmt dataset in the MsigDB database. Using weighted enrichment analysis technology and random combination enrichment detection a thousand times, we calculated the value of FDR and *P* through GSEA. We then visualized the outcomes using R (plyr, ggplot2, grid, grid Extra package) software. Cut-off criteria include gene set size < 15 and >500, nominal *P* value < 0.05, and FDR < 0.25.

### 2.8. Genetic Alteration Analysis

As we logged onto the cBioPortal website (https://www.cbioportal.org/), we opted for the TCGA Pan Cancer Atlas Studies from the quick selection section and went into *DOK5* in order to determine the *DOK5* genetic change characteristics. We observed the change frequency, mutation type, and CNA (copy number change) results of all TCGA tumors in the Cancer Type Summary module.

### 2.9. Clinical Specimens

This study used 24 postoperative tissue samples of patients with GC treated in Changzhou No. 2 People's Hospital from 2019 to 2021. Further, during the operation, the adjacent tissues were collected and stored at 80°C immediately.

### 2.10. Quantitative Real-Time Polymerase Chain Reaction (qRT-PCR) Analysis

The tissues had been preprocessed to extract total RNA. Through the utilization of a PrimeScript RT reagent kit (TaKaRa, Dalian, China), the cDNA was synthesized. Quantitative PCR was carried out with a 7500 real-time PCR system (ABI, Waltham, MA, USA). The PCR primers were synthesized and purchased by Sangon Biotechnology Company (Shanghai, China). *DOK5*: forward: GGTGAAGGGCTGTTTATCTTTC, reverse: TTTTTCACACTCTGTAGCAAGC; GAPDH: forward: CATGTTCCAATATGATTCCAC, reverse: CCTGGAAGATGGTGATG. GAPDH served as an internal control, and fold change was calculated using the 2^−*ΔΔ*CT^ technique.

## 3. Results

### 3.1. *DOK5* mRNA Expression Levels in Different Types of Human Cancers

**Figure 1:**
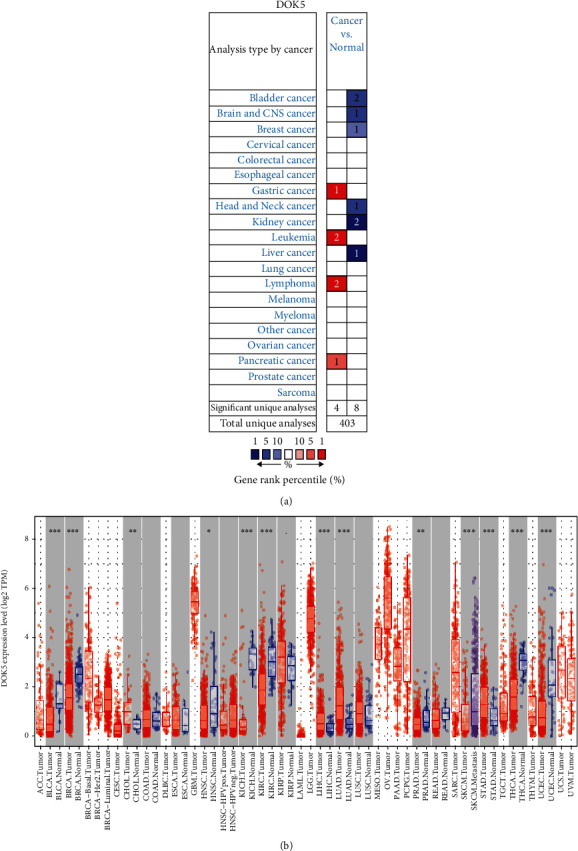
*DOK5* expression levels in different types of human cancers. (a) Increased or decreased *DOK5* in datasets of different cancers compared with normal tissues in the Oncomine database. (b) Human *DOK5* expression levels in different tumor types from TCGA database were determined by TIMER (^∗^*P* < 0.05, ^∗∗^*P* < 0.01, and ^∗∗∗^*P* < 0.001).

In order to determine the difference in the expression of *DOK5* in tumors and normal tissues, we used the Oncomine database to analyze the *DOK5* mRNA levels in normal tissues of different tumors and multiple cancer types. Analysis shows that in contrast to the normal tissues, *DOK5* was better expressed in GC, leukemia, lymphoma, and pancreatic cancer tissues (Figure 1(a)). The in-depth outcomes of *DOK5* expression in varying cancer types have been illustrated in Supplementary Table 1.

For more assessment of the *DOK5* expression in human cancers, we made use of RNA-seq data using several malignant tumors found in TCGA for identifying the *DOK5* expression. In all TCGA tumors, the difference in expression of *DOK5* between the tumor and the adjacent normal tissues has been illustrated in Figure 1(b). The expression of *DOK5* was significantly reduced in bladder urothelial carcinoma (BLCA), breast invasive carcinoma (BRCA), head and neck cancer (HNSC), kidney chromophobe (KICH), kidney renal clear cell carcinoma (KIRC), prostate adenocarcinoma (PRAD), skin cutaneous melanoma (SKCM), thyroid carcinoma (THCA), and uterine corpus endometrial carcinoma (UCEC), compared to adjacent normal tissues. However, *DOK5* expression was significantly increased in cholangiocarcinoma (CHOL), liver hepatocellular carcinoma (LIHC), lung adenocarcinoma (LUAD), and stomach adenocarcinoma (STAD), compared to adjacent normal tissues.

### 3.2. Genetic Change Analysis Data of *DOK5*

**Figure 2:**
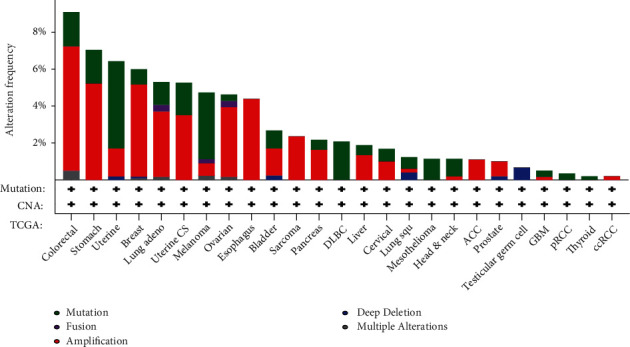
Mutation feature of *DOK5* in different tumors of TCGA. We analyzed the mutation features of *DOK5* for the TCGA tumors using the cBioPortal tool.

In different tumor samples in the TCGA cohort, we observed the genetic changes of *DOK5.* The highest alteration frequency of *DOK5* (>6%) appeared for patients with colorectal tumors, with “amplification” as the primary type. We also observed that the genetic alteration status of *DOK5* was mainly amplified in GC (>4%), which probably explains the changes of *DOK5* in GC tissues at the gene level and gives the foundation for further study (Figure 2).

### 3.3. Effects of *DOK5* on the Prognosis of Different Types of Human Cancers

**Figure 3:**
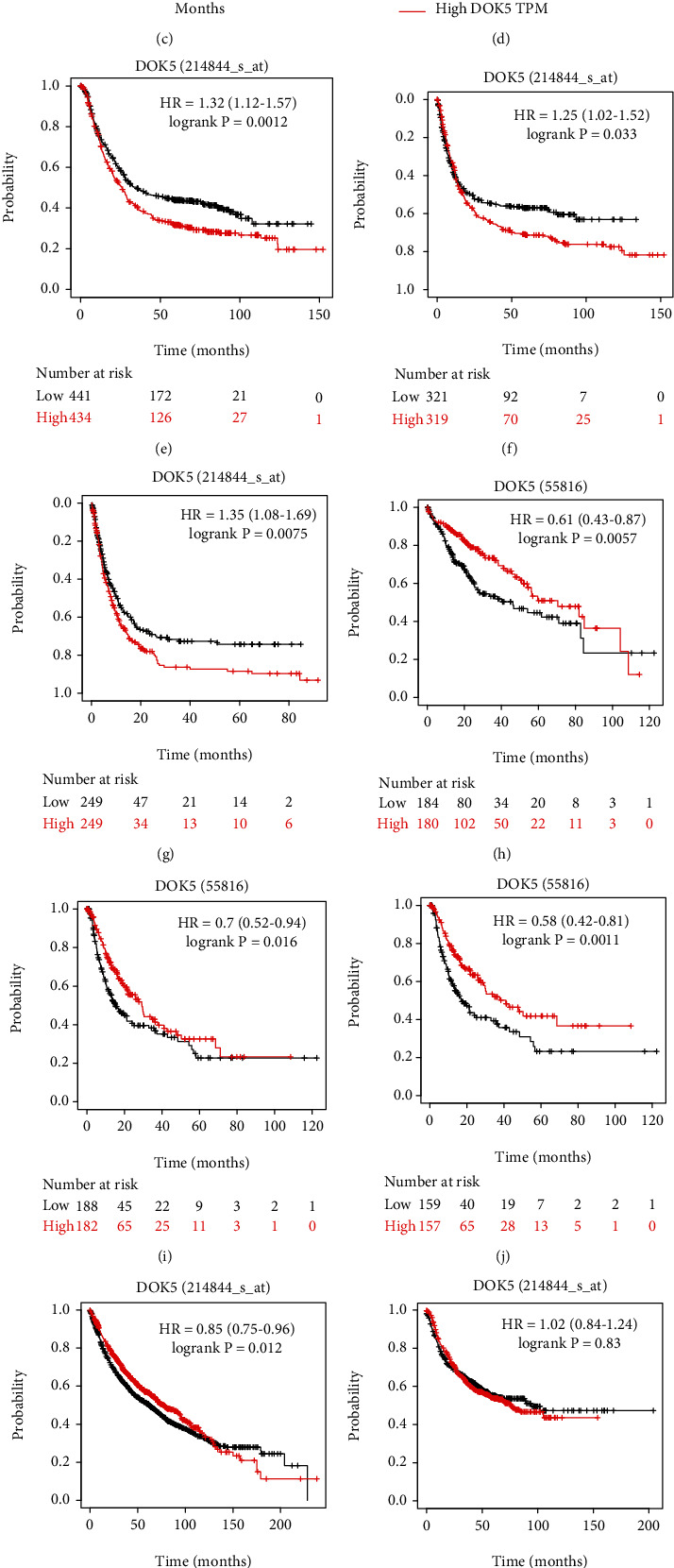
The prognostic value of the mRNA levels of *DOK5* factors in gastric cancer patients (GEPIA and Kaplan-Meier plotter). (a–d) The prognostic value of the mRNA levels of *DOK5* factors in gastric and liver cancer patients analyzed with GEPIA. (e–g) High *DOK5* expression was correlated with bad OS, PFS, and PPS in GC cohorts (*n* = 875, *n* = 640, and *n* = 498). (h–j) Survival curves of OS, PFS, and RFS in the liver cancer cohort (*n* = 364, *n* = 366, and *n* = 313). (k–m) OS, PFS, and PPS survival curves of lung cancer (*n* = 1,925, *n* = 982, and *n* = 344).

In order to study whether the expression of *DOK5* is related to the prognosis of cancer patients, we used GEPIA and Kaplan-Meier plotter to evaluate the effect of *DOK5* expression on survival. Using the data for STAD, LIHC, and LUAD from TCGA in the GEPIA database, we assessed the correlation between differential expressions of *DOK5* and clinical outcomes. Based on results from 381 patients with GC, poorer prognoses in terms of OS and DFS (*P* < 0.05) were associated with higher mRNA expression levels for *DOK5* (Figures 3(a) and 3(b)). However, in liver cancer, different results have emerged. Based on results from 364 patients with liver cancer, poorer prognoses in terms of OS and DFS (*P* < 0.05) were associated with lower mRNA expression levels for *DOK5* (Figures 3(c) and 3(d)).

In order to further study the prognostic potential of *DOK5* in different cancers, the Kaplan-Meier plotter database was used to evaluate the prognostic value of DOK5 based on Affymetrix microarray. It is worth noting that the poor prognosis in GC (OS: HR = 1.32, 95%CI = 1.12 to 1.57, *P* = 0.0012; PFS: HR = 1.02, 95%CI = 1.02 to 1.52, *P* = 0.033; PPS: HR = 1.35, 95%CI = 1.08 to 1.69, *P* = 0.0075) was shown to correlate with higher *DOK5* expression (Figures 3(e)–3(g)). Poor prognosis is associated with low *DOK5* expression in liver cancer (OS: HR = 0.61, 95%CI = 0.43 to 0.87, *P* = 0.0057; PFS: HR = 0.7, 95%CI = 0.52 to 0.94, *P* = 0.016; RFS: HR = 0.58, 95%CI = 0.58 to 0.81, *P* = 0.0011; Figure 3(h)–3(j)). Sawant et al.'s studies have also shown that high *DOK5* expression is associated with poor prognosis in liver cancer [9]. The expression of DOK5 was also correlated with the patients' survival in the lung cancer (OS: HR = 0.85, 95% CI = 0.75 to 0.96, *P* = 0.0126; PFS: HR = 1.02, 95% CI = 0.84 to 1.24, *P* = 0.83; PPS: HR = 0.86, 95% CI = 0.67 to 1.11, *P* = 0.25; Figures 3(k)–3(m)). Conversely, *DOK5* expression was not related with PFS and PPS in lung cancer (Figures 3(l) and 3(m)). These results suggest that *DOK5* expression is of prognostic significance in GC, liver cancer, and lung cancer.

### 3.4. Expression and Clinical Features of *DOK5* in Patients with Gastric Cancer

We analyzed the influence of *DOK5* expression on different types of clinical patients using the Kaplan-Meier plotter database (Table 1). High *DOK5* expression correlated with both poorer OS and PPS in stage 3 patients (OS: HR = 1.54, *P* < 0.05; PFS: HR = 1.77, *P* < 0.01), stage T2 patients (OS: HR = 1.87, *P* < 0.01; PFS: HR = 1.66, *P* < 0.05), stage M0 patients (OS: HR = 1.8, *P* < 0.001; PFS: HR = 1.71, *P* < 0.001), intestinal patients (OS: HR = 1.95, *P* < 0.001; PFS: HR = 1.73, *P* < 0.01), diffuse patients (OS: HR = 1.63, *P* < 0.01; PFS: HR = 1.67, *P* < 0.01), and HER2-negative patients (OS: HR = 1.47, *P* < 0.001; PFS: HR = 1.35, *P* < 0.05). It is worth noting that among patients with lymph node metastasis, patients with high *DOK5* expression have a poorer prognosis (OS: stage N1, HR = 2.43, *P* < 0.001; stage N2, HR = 1.78, *P* < 0.05; stage N3, HR = 1.88, *P* < 0.05; stage N1+2+3, HR = 1.97, *P* < 0.001. PFS: stage N1, HR = 2.49, *P* < 0.001; stage N2, HR = 1.56, *P* < 0.05; stage N1+2+3, HR = 1.9, *P* < 0.001). However, in stages 1, 2, T3, T4, N0, M1, and HER2-positive patients, *DOK5* expression was not related to OS and PFS. The above data shows that according to the clinical characteristics of patients with GC, *DOK5* expression is related to patients with GC lymph node metastasis.

### 3.5. *DOK5* Expression in Patients with STAD

**Figure 4:**
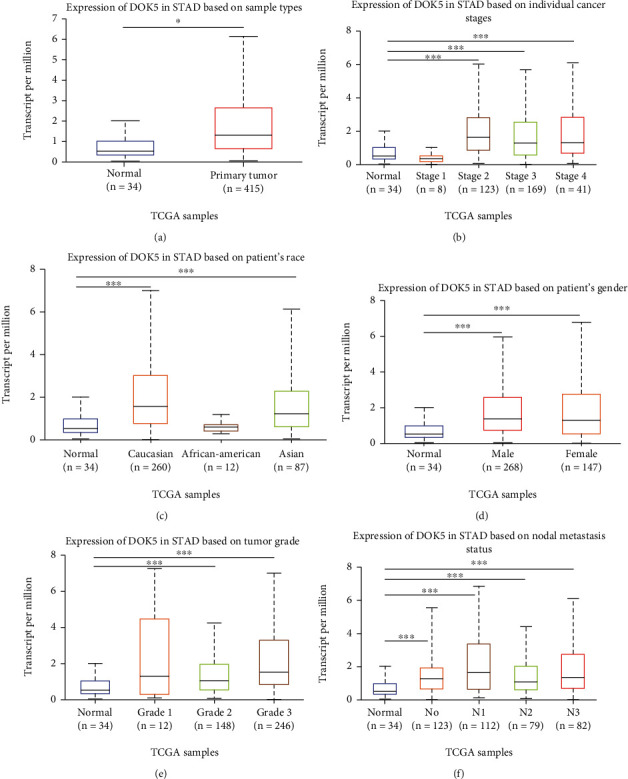
*DOK5* expression in subgroups of patients with STAD (UALCAN database). Relative expression of *DOK5* in (a) STAD and normal samples; (b) normal individuals and patients with STAD at different stages; (c) normal individuals and Caucasian, African American, and Asian patients with STAD; (d) male and female normal individuals and patients with STAD; (e) normal individuals and STAD patients of different tumor grades; and (f) nodal metastasis status of patients with STAD (STAD: stomach adenocarcinoma; ^∗^*P* < 0.05, ^∗∗^*P* < 0.01, and ^∗∗∗^*P* < 0.001).

In the UALCAN database, we further analyzed various clinicopathological characteristics of TCGA-STAD specimens and it was found that compared with normal tissues, the expression level of *DOK5* mRNA was higher in STAD tissues (Figure 4(a)). The expression of *DOK5* in patients with STAD of different stages (2-4) was significantly higher than that of normal controls (Figure 4(b)). In addition, in the assessment made on the basis of race, the expression of *DOK5* in patients with STAD was essentially high in contrast to that in the control group (specifically in Caucasians and Asians (Figure 4(c)) and sex (Figure 4(d))). The expression of *DOK5* in patients having different grades (1, 3) of STAD was significantly more than that of the normal controls (Figure 4(e)). In the end, in patients with lymph node metastasis, *DOK5* expression level is also higher (Figure 4(f)). Hence, the expression level of *DOK5* is expected to be a potential diagnostic indicator for tumor staging in patients with GC.

### 3.6. *DOK5* Expression Is Associated with Immune Cell Infiltration in GC

**Figure 5:**
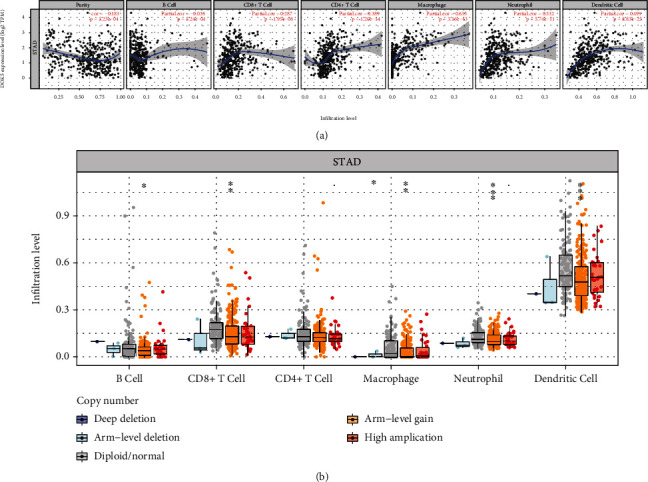
Correlation analysis of *DOK5* expression and infiltration levels of immune cells in GC tissues using the TIMER database. (a) *DOK5* expression is significantly negatively associated with tumor purity including B cell and has significant positive correlations with infiltrating levels of CD8+ T cells, CD4+ T cells, macrophages, neutrophils, and dendritic cell (*n* = 415). (b) CNA of DOK5 had significant correlations with immune infiltration cells including B cells, CD8+ T cells, macrophage, neutrophil, and dendritic cell.

The number and active state of tumor-infiltrating lymphocytes can determine the survival time of some patients with cancer [10]. Therefore, we made use of the TIMER database for the identification of the relationship shared by the *DOK5* expression and infiltrating immune cells in 32 cancers, including GC. The results showed that in 32 tumor types, *DOK5* expression was crucially related to CD4+ T cells, CD8+ T cells, neutrophils, macrophages, and dendritic cells (Figure 5(a)). Furthermore, we also explored the link shared by SCNA (somatic copy number alteration) of *DOK5* gene and the level of GC tumor invasion. It is worth noting that the results show that the CNA of *DOK5* is significantly related to the infiltration level of CD8+ T cells, B cells, neutrophils, macrophages, and dendritic cells (Figure 5(b)).

### 3.7. Correlation between Immune Marker Sets and *DOK5* Expression

**Figure 6:**
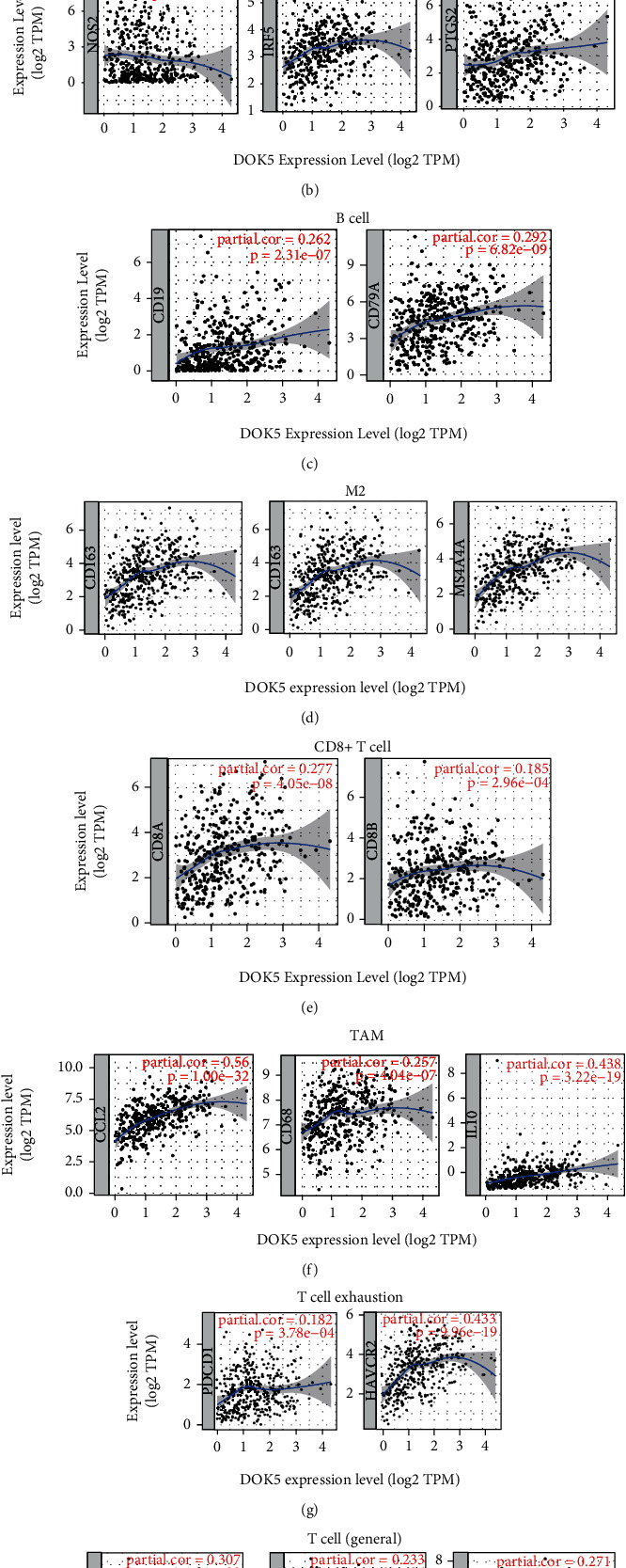
*DOK5* expression correlated with macrophage polarization in STAD (stomach adenocarcinoma). Markers include CD86 and CSF1R of monocytes (a); NOS2, IRF5, and PTGS2 of M1 macrophages (b); CD19 and CD79A of B cell (c); CD163, VSIG4, and MS4A4A of M2 macrophages (d); CD8A and CD8B of CD8+ T cell (e); CCL-2, CD68, and IL10 of TAMs (f); PDCD1 and HAVCR2 of T cell exhaustion (g); and CD3D, CD3E, and CD2 of T cell (general) (h).

In exploring the relationship shared by *DOK5* as well as various immune infiltrating cells, using GEPIA and TIMER databases, we paid attention to the relationship shared by various immune cell marker sets and *DOK5* in GC tissues. In STAD, we examined the link shared by *DOK5* expression and varying immune cells, like CD8+ T cells, B cells, T cells (general), TAMs, M1 macrophages, M2 macrophages, monocytes, neutrophils, NK cells, and DCs (Table 2 and Figures 6(a)–6(h)). We examined T cells with different functions, involving Th1 cells, Th2 cells, Tfh cells, Th17 cells, Treg cells, and exhausted T cells. The results showed that in STAD, the expression level of *DOK5* is related to most immune marker sets of different T cells and various immune cells (Table 2).

It is worth noting that in GC, we explored the levels of expressions of most marker groups of monocytes; TAMs and M2 macrophage held a significant correlation with *DOK5* expression (Table 2). We showed that CD86 and CSF1R of monocytes; IRF5 and PTGS2 of M1 macrophages; CD19 and CD79A of B cell; CD163, VSIG4, and MS4A4A of M2 macrophages; CD8A and CD8B of CD8+ T cell; CCL2, CD68, and IL10 of TAM; PDCD1 and HAVCR2 of T cell exhaustion; and CD3D, CD3E, and CD2 of T cell (general) are crucially linked to *DOK5* expression in GC (*P* < 0.001; Figures 6(a)–6(h)). We further examined the link shared by *DOK5* expression with monocytes markers and TAM markers in the GEPIA database. Correlation outcomes of *DOK5* with monocyte markers and TAM markers matched the TIMER (Table 3). The above outcomes suggest that in GC, *DOK5* may be related to the regulation of macrophage polarization.

*DOK5* expression was positively correlated with dendritic cell infiltration in GC; for example, HLA-DPB1, HLA-DRA, HLA-DQB1, NRP1, CD1C, and ITGAX are also related to the expression of *DOK5*. The above outcomes also disclosed the proximate link shared by *DOK5* and dendritic cell infiltration. In terms of Treg cells, *DOK5* is positively correlated with FOXP3, CCR8, and TGFB1 in GC. In tumors, dendritic cells can promote metastasis through the reduction of cytotoxicity of CD8+ T cells and increasing Treg cells [11]. Moreover, we explored a strong link shared by *DOK5* and T cell exhaustion and Treg, for instance, CCR8, FOXP3, STAT5B, TGFB1, PDCD1, LAG3, CTLA4, and HAVCR2 (Table 2). In Treg cells, FOXP3 can inhibit cytotoxic T cells from attacking tumor cells [12]. It is worth noting that HAVCR2, as a key gene regulating T cell exhaustion, is strongly correlated with the high expression of *DOK5*, highlighting that *DOK5* holds an essential part in T cell exhaustion. In summary, the above results further indicate that *DOK5* is specifically linked to immune infiltrating cells in GC, pointing towards that *DOK5* has a crucial immune role in the microenvironment of GC.

### 3.8. Gene Set Enrichment Analysis of *DOK5*

**Figure 7:**
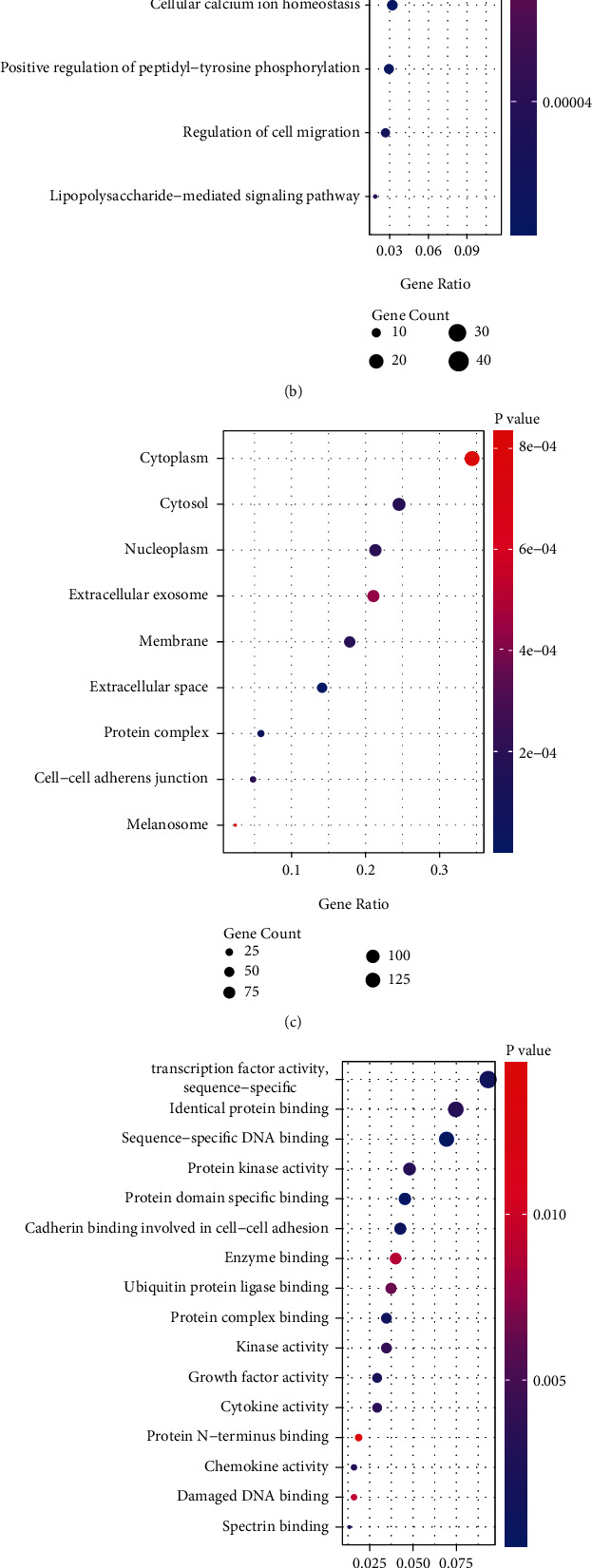
GSEA analysis, GO, and KEGG enrichment analyses of *DOK5* (differentially expressed genes). GO: Gene Ontology; KEGG: Kyoto Encyclopedia of Genes and Genomes. Verification of upregulation of DOK5 in GC by qRT-PCR. A combined enrichment plot has been provided with the analysis of the enrichment of the gene series, including the enrichment fraction and gene series (it is mainly immune-related pathway) (a). Coexpression network of *DOK5* is performed by function enrichment analysis by GO in GC (b–d). Coexpression network of *DOK5* is performed by function enrichment analysis by KEGG in GC (e). Verification of upregulation of *DOK5* in GC by qRT-PCR (^∗^*P* < 0.05, ^∗∗^*P* < 0.01, and ^∗∗∗^*P* < 0.001) (f).

According to TCGA information, the ability to search for *DOK5* and its related symbol transmission is realized using GSEA. As per NES, nominal *P* value, and FDR *q* value, fundamentally advanced flagging pathways had been elected. In this study, 19 signaling measures were differentially enhanced in the profoundly communicated phenotypes of *DOK5*. We discovered that most of these pathways are immune-related and involve cell adhesion molecules (CAMS), gap junction, complement and coagulation cascades, ECM receptor interaction, cytokine-cytokine receptor interaction, hedgehog signaling pathway, and leukocyte transendothelial migration (Figure 7(a)).

### 3.9. Functional Enrichment Analysis of *DOK5* Gene

To understand the biological properties of *DOK5* completely, we carried out GO and KEGG analyses. On the basis of the outcomes of DAVID's research, we explored biologically enriched genes that are positively linked to the *DOK5* expression levels. In GO analysis, the two biological processes contained by genes that are positively associated with *DOK5* expression are as follows: immune response and the inflammatory response. Nine cellular components have been included in these coexpressed genes: cytoplasm, cytosol, nucleoplasm, extracellular exosome, membrane, extracellular space, protein complex, cell-cell adherents' junction, and melanosome. Moreover, these coexpressed genes have three main molecular functions: sequence-specific DNA binding, identical protein binding and protein kinase activity, and transcription factor activity. Genes positively correlated with *DOK5* expression in KEGG pathway analysis were as follows: cytokine-cytokine receptor interaction, transcriptional misregulation in cancer, TNF signaling pathway, malaria, and Chagas disease (American trypanosomiasis) (Figures 7(b)–7(e)).

### 3.10. qRT-PCR Experiments Show That *DOK5* Expression Is Upregulated in Gastric Cancer

In order to further confirm that the expression level of *DOK5* in GC tissues is higher than that in adjacent tissues, we used qRT-PCR technology to reveal the expression of *DOK5* at the transcription level. The results showed that compared with adjacent nontumor tissues, the expression level of *DOK5* mRNA in GC tissues was significantly increased (Figure 7(f)).

## 4. Discussion

GC is a tumor of the digestive system with high morbidity and mortality worldwide [13]. At present, surgery can be said to be the most efficient treatment for early GC, and 90% of patients will have a good prognosis [14]. However, resulting from the lack of early diagnosis, many patients are diagnosed at advanced stages, for example, about 65% of patients with stage 3 and stage 4 tumors, and nearly 85% of patients have lymph node metastasis [15]. In addition, treatment resistance often appears during the treatment of advanced GC. Immunomodulation has been applied to various types of cancer in preclinical models and has achieved good results [16]. It is of great significance for exploring an efficient method for the early diagnosis, as well as treatment of GC.

In this study, the Oncomine database and the TCGA database analyses showed that *DOK5* expression was higher in GC than in normal tissues; these complied with the results of related research reports [17]. It has also been studied in breast cancer, liver cancer, and colorectal cancer, and *DOK5* gene expression in cancer tissues is higher than that in normal tissues [18]. The receptor, tyrosine kinase c-Ret, has been found to be an oncogenic mutation in patients with multiple endocrine tumors and cancer syndromes with familial medullary thyroid carcinoma, and *DOK5* can be directly associated with Y1062 of c-Ret, thereby enhancing the effect of c-Ret [3]. Our research shows that *DOK5* is likely to be an oncogene that holds a crucial part in the development and occurrence of cancer. Moreover, in patients with stomach cancer, the *DOK5* expression level is also significantly different in different pathological stages, tumor differentiation, and T stages. Therefore, we further studied the link shared by the expression of *DOK5* and clinicopathological boundaries and found that the *DOK5* expression level had been associated with lymph node metastasis and T stage. Moreover, for patients with lymph node metastasis, patients with high *DOK5* expression have a poor prognosis, while patients with low *DOK5* expression have a better prognosis. The pathological, T, N, and M stages are linked to the prognosis of GC patients. Our research shows that *DOK5* is related to the survival and prognosis of GC patients; therefore, this suggests that *DOK5* may be a specific marker of gastric cancer. Nineteen signal pathways were enriched by GSEA to analyze the signal pathway of *DOK5* in GC. Melanoma, basal cell carcinoma, and hematopoietic cell lineage all proved that *DOK5* influences the progression as well as the occurrence of tumors. In addition, we use the research method of Dr. Sun and his colleagues as a reference to conduct correlation analysis on *DOK5* [19–21].

Recently, the role of the immune system in the development, as well as occurrence of cancer, was given more attention [22, 23]. Exploring the tumor microenvironment is a new hot research field for tumor diagnosis and treatment [24]. Focal adhesion was found to affect cell migration [25, 26]. Studies have shown that focal adhesion is linked to several biological pathways like cell differentiation, cell proliferation, and cell survival [27]. Simultaneously, focal adhesions are also related to the invasion of cancer cells [28]. Studies have shown that in the tumor microenvironment, ECM receptor interaction plays a significant part in tumor metastasis and recurrence [29]. Tumor angiogenesis and tumor local invasion along with distant metastasis are closely associated to the CAM pathway [30]. Studies have shown that the expression of the CAM pathway can help identify the prognosis of tumors and is expected to turn into a new target for GC treatment. Moreover, the cytokine-cytokine receptor interaction signaling pathway plays a crucial role in cancer pathogenesis [31]. There are also research results showing that the restoration of gap junction will affect the growth of tumor cells and the differentiation of tumor tissues [32]. Therefore, the role of gap junctions can affect the efficacy of antitumor drugs, thereby providing new targets for tumor treatment [33]. There are related research reports on transcription regulation errors playing very important roles in the development of tumors [34]. Tumor necrosis factor (TNF) has a main part in the development of inflammation, cell proliferation, and cell death [35]. For a long time in the past, the TNF family signaling pathway has been a double-edged sword in the process of tumor occurrence and clearance [36]. In the pathogenesis of oral squamous cell carcinoma, TNF-*α* can regulate EMT through the MAPK signaling pathway to promote cancer cell invasion and metastasis, and *DOK5* also involved in MAPK (mitogen-activated protein kinase) signal pathway activation [37]. Therefore, we have reason to speculate that *DOK5* may also participate in tumor progression through the MAPK pathway. Therefore, the above results explain the high expression of *DOK5* and the high level of immune cell infiltration can reduce the survival rate of patients with GC. These results may bring new methods for the immunotherapy of patients with GC.

Without doubt, after the above results and demonstrations, we have reason to believe that *DOK5* is linked to the GC development as well as occurrence. The high expression of *DOK5* further impairs the prognosis of patients with GC of by participating in immune-related mechanisms. However, our research also has certain limitations. Our data comes from different databases, and the information in each database may have some differences. Fortunately, through mutual verification of the different databases, we finally got our results.

## 5. Conclusions

Using multiple database verifications, we found that *DOK5* is an oncogene of GC. Hence, *DOK5* can reduce the prognostic effect of GC in patients through immune response. *DOK5* is expected to become a new target for GC treatment and provide a new direction for GC treatment.

## Figures and Tables

**Table 1:** Kaplan-Meier plotter was used to analyze the correlation between *DOK5* mRNA expression and different clinicopathological factors in gastric cancer.

Clinicopathological factors	Overall survival			Progression-free survival		
	*N*	Hazard ratio	*P* value	*N*	Hazard ratio	*P* value
Sex						
Female	244	1.47 (1.03-2.08)	∗	201	1.46 (1-2.13)	0.051
Male	566	1.28 (1.03-1.59)	∗	437	1.08 (0.85-1.37)	0.55
Stage						
1	69	0.63 (0.23-1.71)	0.36	60	0.66 (0.22-2)	0.46
2	145	1.74 (0.93-3.25)	0.077	131	1.5 (0.8-2.79)	0.2
3	319	1.54 (1.15-2.05)	∗	186	1.77 (1.21-2.58)	∗∗
4	152	1.5 (1.02-2.21)	∗	141	1.41 (0.96-2.07)	0.083
Stage T						
1	14	—	—	14	—	—
2	253	1.87 (1.21-2.9)	∗∗	239	1.66 (1.09-2.54)	∗
3	208	1.27 (0.9-1.8)	0.17	204	1.22 (0.87-1.7)	0.24
4	39	1.59 (0.69-3.62)	0.27	39	2.49 (1.14-5.47)	∗
Stage N						
0	76	1.04 (0.44-2.47)	0.92	72	1.21 (0.52-2.8)	0.66
1	232	2.43 (1.57-3.75)	∗∗∗	222	2.49 (1.64-3.79)	∗∗∗
2	129	1.78 (1.13-2.81)	∗	125	1.56 (1.01-2.41)	∗
3	76	1.88 (1.1-3.22)	∗	76	1.59 (0.93-2.72)	0.085
1+2+3	437	1.97 (1.51-2.58)	∗∗∗	423	1.9 (1.47-2.47)	∗∗∗
Stage M						
0	459	1.8 (1.36-2.39)	∗∗∗	443	1.71 (1.3-2.24)	∗∗∗
1	58	1.76 (0.98-3.17)	0.0573	56	1.4 (0.77-2.53)	0.27
Lauren classification						
Intestinal	336	1.95 (1.41-2.7)	∗∗∗	263	1.73 (1.2-2.47)	∗∗
Diffuse	248	1.63 (1.16-2.3)	∗∗	231	1.67 (1.18-2.37)	∗∗
Mixed	33	2.02 (0.72-5.71)	0.1743	28	1.67 (0.6-4.68)	0.32
Differentiation						
Poorly differentiated	165	1.19 (0.8-1.78)	0.39	121	1.35 (0.85-2.15)	0.2
Moderately differentiated	67	1.41 (0.73-2.69)	0.3	67	1.58 (0.85-2.96)	0.15
Well differentiated	32	2.45 (1.01-5.95)	∗	5	—	—
HER2 status						
HER2 negative	532	1.47 (1.17-1.85)	∗∗∗	408	1.35 (1.04-1.75)	∗
HER2 positive	343	1.16 (0.9-1.51)	0.26	232	1.24 (0.9-1.71)	0.19

^∗^ *P* < 0.05, ^∗∗^*P* < 0.01, and ^∗∗∗^*P* < 0.001.

**Table 2:** Correlation analysis between *DOK5* and related genes and markers of immune cells in TIMER.

Description	Gene markers	STAD			
		None		Purity	
		Core	*P*	Core	*P*
CD8+ T cell	CD8A	0.319	∗∗∗	0.277	∗∗∗
	CD8B	0.216	∗∗∗	0.185	∗∗∗
T cell (general)	CD3D	0.286	∗∗∗	0.233	∗∗∗
	CD3E	0.318	∗∗∗	0.271	∗∗∗
	CD2	0.348	∗∗∗	0.307	∗∗∗
B cell	CD19	0.288	∗∗∗	0.262	∗∗∗
	CD79A	0.333	∗∗∗	0.292	∗∗∗
Monocyte	CD86	0.464	∗∗∗	0.426	∗∗∗
	CD115 (CSF1R)	0.55	∗∗∗	0.523	∗∗∗
TAM	CCL2	0.594	∗∗∗	0.56	∗∗∗
	CD68	0.291	∗∗∗	0.257	∗∗∗
	IL10	0.457	∗∗∗	0.438	∗∗∗
M1 macrophage	INOS (NOS2)	-0.065	0.183	-0.09	0.08
	IRF5	0.301	∗∗∗	0.291	∗∗∗
	COX2 (PTGS2)	0.216	∗∗∗	0.217	∗∗∗
M2 macrophage	CD163	0.448	∗∗∗	0.416	∗∗∗
	VSIG4	0.492	∗∗∗	0.47	∗∗∗
	MS4A4A	0.526	∗∗∗	0.501	∗∗∗
Neutrophils	CD66b (CEACAM8)	-0.034	∗∗∗	-0.029	∗∗∗
	CD11b (ITGAM)	0.496	∗∗∗	0.482	∗∗∗
	CCR7	0.398	∗∗∗	0.357	∗∗∗
Natural killer cell	KIR2DL1	0.142	∗∗	0.124	∗
	KIR2DL3	0.079	0.11	0.043	0.405
	KIR2DL4	-0.017	0.73	-0.052	0.316
	KIR3DL1	0.159	∗∗	0.134	∗∗
	KIR3DL2	0.175	∗∗∗	0.14	∗∗
	KIR3DL3	-0.107	∗	-0.102	∗
	KIR2DS4	0.059	0.229	0.04	0.437
Dendritic cell	HLA-DPB1	0.394	∗∗∗	0.35	∗∗∗
	HLA-DQB1	0.205	∗∗∗	0.154	∗∗
	HLA-DRA	0.285	∗∗∗	0.241	∗∗∗
	HLA-DPA1	0.324	∗∗∗	0.28	∗∗∗
	BDCA-1 (CD1C)	0.455	∗∗∗	0.431	∗∗∗
	BDCA-4 (NRP1)	0.627	∗∗∗	0.615	∗∗∗
	CD11c (ITGAX)	0.459	∗∗∗	0.432	∗∗∗
Th1	T-bet (TBX21)	0.301	∗∗∗	0.264	∗∗∗
	STAT4	0.358	∗∗∗	0.327	∗∗∗
	STAT1	0.033	0.507	0.012	0.815
	IFN-*γ* (IFNG)	0.064	0.196	0.0036	0.482
	TNF-*α* (TNF)	0.158	∗∗	0.109	∗
Th2	GATA3	0.363	∗∗∗	0.337	∗∗∗
	STAT6	0.154	∗∗	0.148	∗∗
	STAT5A	0.384	∗∗∗	0.383	∗∗∗
	IL13	0.179	∗∗∗	0.207	∗∗∗
Tfh	BCL6	0.416	∗∗∗	0.396	∗∗∗
	IL21	0.116	∗	0.0096	0.063
Th17	STAT3	0.336	∗∗∗	0.337	∗∗∗
	IL17A	-0.148	∗∗	-0.158	∗∗
Treg	FOXP3	0.33	∗∗∗	0.287	∗∗∗
	CCR8	0.397	∗∗∗	0.384	∗∗∗
	STAT5B	0.462	∗∗∗	0.457	∗∗∗
	TGF*β* (TGFB1)	0.576	∗∗∗	0.552	∗∗∗
T cell exhaustion	PD-1 (PDCD1)	0.221	∗∗∗	0.182	∗∗∗
	CTLA4	0.17	∗∗∗	0.125	∗
	LAG3	0.179	∗∗∗	0.136	∗∗
	TIM-3 (HAVCR2)	0.456	∗∗∗	0.433	∗∗∗
	GZMB	0.119	∗	0.066	0.203

STAD: stomach adenocarcinoma; TAM: tumor-associated macrophage; Th: T helper cell; Tfh: follicular helper T cell; Treg: regulatory T cell; Cor: *R* value of Spearman's correlation; None: correlation without adjustment; Purity: correlation adjusted by purity (^∗^*P* < 0.05, ^∗∗^*P* < 0.01, and ^∗∗∗^*P* < 0.001).

**Table 3:** Correlation analysis between *DOK5* and marker genes of immune cells in GEPIA.

Description	Gene markers	STAD			
		Tumor		Normal	
		*R*	*P*	*R*	*P*
Monocyte	CD86	0.26	∗∗∗	−0.11	0.53
	CD11b	0.26	∗∗∗	0.57	∗∗∗
Neutrophils	CCR7	0.19	∗∗∗	−0.14	0.42
TAM	CD68	0.18	∗∗∗	−0.41	∗
	IL-10	−0.024	0.63	0.099	0.57
Th1	IFN-*γ* (IFNG)	−0.042	0.4	−0.058	0.74
	STAT1	−0.083	0.095	0.18	0.3
	T-bet (TBX21)	0.15	∗	−0.052	0.76
	TNF-*α* (TNF)	0.11	∗	−0.15	0.37
Th2	STAT6	0.058	0.24	0.48	∗∗
Treg	CCR8	0.18	∗∗∗	−0.27	0.11
	STAT5B	0.3	∗∗∗	0.77	∗∗∗
	TGF-*β* (TGFB1)	0.45	0	0.066	0.7
T cell exhaustion	CTLA4	−0.049	0.33	−0.15	0.39
	PD-1 (PDCD1)	0.03	0.55	−0.16	0.34
	TIM-3 (HAVCR2)	0.22	∗∗∗	0.02	0.91

^∗^ *P* < 0.05, ^∗∗^*P* < 0.01, and ^∗∗∗^*P* < 0.001.

## Data Availability

All data were acquired from public databases, including TCGA, Oncomine, GEPIA, Kaplan-Meier plotter, cBioPortal, TIMER, and UALCAN database.

## Supplementary Materials

Supplementary Materials Supplementary Table 1: *DOK5* expression in cancers versus normal tissue in Oncomine database.
