# Supplementary material for: DOK5 as a Prognostic Biomarker of Gastric Cancer Immunoinvasion: A Bioinformatics Analysis
Source: Biomed Res Int. 2022 Jan 5;2022:9914778. doi: 10.1155/2022/9914778 (PMC8754673; doi:10.1155/2022/9914778)
Supplement: Supplementary Materials — Supplementary Table 1: DOK5 expression in cancers versus normal tissue in Oncomine database. [file 9914778.f1.doc]

**Supplementary Table 1. DOK5 expression in cancers verus normal tissue in oncomine database.**

| **Cancer** | **Cancer type** | ***P-value*** | **Fold change** | **Rank (%)** | **Sample** | **Reference (PMID)** |
| --- | --- | --- | --- | --- | --- | --- |
| Bladder | Superficial Bladder Cancer | 1.56E-8 | -2.115 | 2% | 42 | [15173019](https://www.ncbi.nlm.nih.gov/pubmed/15173019) |
|  | Infiltrating Bladder Urothelial Carcinoma | 1.69E-6 | -2.002 | 3% | 27 | [15173019](https://www.ncbi.nlm.nih.gov/pubmed/15173019) |
| Brain and CNS | Glioblastoma | 7.96E-10 | -4.193 | 3% | 25 | [16697959](https://www.ncbi.nlm.nih.gov/pubmed/16697959) |
| Breast | Invasive Ductal Breast Carcinoma | 1.79E-22 | -2.095 | 8% | 450 | TCGA |
| Gastric | Gastric Cancer | 1.06E-5 | 2.263 | 1% | 27 | [21132402](https://www.ncbi.nlm.nih.gov/pubmed/21132402) |
| Head and Neck | Tongue Squamous Cell Carcinoma | 9.03E-6 | -2.291 | 5% | 57 | [19138406](https://www.ncbi.nlm.nih.gov/pubmed/19138406) |
| Kidney | Chromophobe Renal Cell Carcinoma | 1.86E-6 | -5.664 | 1% | 9 | [20440404](https://www.ncbi.nlm.nih.gov/pubmed/20440404) |
|  | Renal Oncocytoma | 4.80E-5 | -4.826 | 1% | 9 | [20440404](https://www.ncbi.nlm.nih.gov/pubmed/20440404) |
| Leukemia | Acute Adult T-Cell Leukemia/Lymphoma | 8.53E-11 | 8.511 | 1% | 28 | [16909099](https://www.ncbi.nlm.nih.gov/pubmed/16909099) |
|  | Chronic Adult T-Cell Leukemia/Lymphoma | 3.86E-8 | 5.194 | 1% | 25 | [16909099](https://www.ncbi.nlm.nih.gov/pubmed/16909099) |
| Liver | Hepatocellular Adenoma | 9.63E-13 | -2.376 | 1% | 72 | [12058060](https://www.ncbi.nlm.nih.gov/pubmed/12058060) |
| Lymphoma | Acute Adult T-Cell Leukemia/Lymphoma | 8.53E-11 | 8.511 | 1% | 28 | [16909099](https://www.ncbi.nlm.nih.gov/pubmed/16909099) |
|  | Chronic Adult T-Cell Leukemia/Lymphoma | 3.86E-8 | 5.194 | 1% | 25 | [16909099](https://www.ncbi.nlm.nih.gov/pubmed/16909099) |
| Pancreatic | Pancreatic Ductal Adenocarcinoma | 6.90E-11 | 3.042 | 3% | 78 | [19260470](https://www.ncbi.nlm.nih.gov/pubmed/19260470)  [28881803](https://www.ncbi.nlm.nih.gov/pubmed/28881803) |
